# Determinants of Continuance Intention to Use Health Apps among Users over 60: A Test of Social Cognitive Model

**DOI:** 10.3390/ijerph181910367

**Published:** 2021-10-01

**Authors:** Eunhye Kim, Semi Han

**Affiliations:** School of AI Healthcare, CHA University, Pocheon 11160, Korea; eunhyekim@cha.ac.kr

**Keywords:** health apps, older users, continuance intention to use, self-management, social cognitive theory

## Abstract

Promoting healthy behavior among seniors is important in reducing the burden of care and healthcare expenses in a rapidly aging society. Health apps can play an important role in health promotion for older adults, but the level of user retention in health apps is low. To increase continued health app use among older adults, this study examined the factors influencing older users’ continuance intentions to use health apps. The research model was developed based on the social cognitive theory of health behavior, which integrates cognitive, environmental, and behavioral perspectives. To test the research model, an anonymous online survey was conducted among respondents aged 60 to 79 years who were using health apps. The measurement items in the questionnaire were developed based on validated scales from the literature. A total of 250 samples were analyzed. The assessment of the measurement model indicates that the reliability and validity of the items are satisfactory. The results of testing the structural model illustrate the determinants of health app continuance intention: health technology self-efficacy, self-evaluative outcome expectations, self-regulation, and privacy risk. The interrelationships among determinants are also investigated. Theoretical and practical implications are suggested to encourage older adults’ continued health app use and promote their health behavior over the long term.

## 1. Introduction

While the aging population is rapidly growing, the number of people who can take care of older adults and the budget to care for them is limited. Therefore, the dependent generation’s burden of supporting and caring for seniors is increasing. To reduce the burden of caring for older adults, it is necessary to manage their health in their daily lives and prevent chronic disease. Effective health management for older adults can not only reduce the burden of care for the dependent generation but also improve older adults’ quality of life by enabling them to enjoy an active and independent old age. Healthy habits and behaviors such as regular exercise, good sleep patterns, and healthy eating are important in the successful management and maintenance of older adults’ health. For example, it is well known that regular exercise can reduce the risk of death and chronic disease and enhance quality of life [1]. For seniors, in particular, regular exercise is critical in ensuring better health, but many older people do not participate in regular and sufficient exercise [2]. To encourage healthy behavior, such as regular exercise, among older people, self-management of healthcare is important [3]. However, although the importance of self-management of healthy habits is increasing, it is difficult to persuade people to engage in such self-management. Self-management of health cannot be achieved solely by deciding to engage in such behavior, and several psychological subfunctions—including motivation, self-efficacy, and self-regulation—are necessary drivers of self-management behavior [3]. Thus, the role of a personal coach who can motivate older adults to self-manage their health, help set goals, and monitor goal achievement is important. However, it would be prohibitively expensive to find a personal coach for each older adult.

With the recent development of digital technology, consumer health technology is attracting attention as a useful and effective tool for the self-management of health and as a successful and efficient complement to a personal coach. Health technology enables people to be more active in their health management [4,5]. Among the various types of health technology, health apps are the most prevalent for general users. On such apps, users (including older adults) expect to receive personalized health information and advice, change their health habits and behaviors, and improve their health outcomes [6,7]. It has been proven that health apps can enhance the effectiveness of health interventions [8]. However, these positive outcomes are realized only when users use the app continuously, as opposed to initial adoption or short usage. In reality, health app users often stop using such apps: user retention in health apps is low [9]. Thus, the positive outcomes of health apps may not be maximized. Due to the importance of continuing use of health technology, including health apps, continuous use behavior has been researched among general users; however, insufficient research targeting older adults has been conducted [10]. Therefore, it is critical to investigate older adults’ usage behavior toward health apps to provide better health interventions for them. Since health apps represent the convergence of technology and health services, it is important to understand both technology use and health behavior. Furthermore, even the same health technology often elicits different usage behavior from different individuals. This means that usage behavior can be affected not only by the characteristics of the health technology but also by users’ personal characteristics, such as cognition [8]. Cognition variables are more proximal to explaining behavior and are more open to reflecting changes than other variables, such as demographics [1]. Therefore, this research attempted to identify the factors affecting older adults’ intentions to continue use of health apps, focusing on variables related to cognition. We will introduce social cognitive theory of health behavior, which is employed as the theoretical background of this study among various cognition related theories. Next, we will introduce SCT-based prior research which uses SCT to predict and explain health behavior and health technology usage behavior. Based on these theoretical backgrounds, we then propose our research model.

### 1.1. Research Background 

There are various social cognitive theories, including the health belief model [11], the theory of planned behavior [12], protection motivation theory [13], and the transtheoretical model [14]. Here, we employed the social cognitive theory (SCT) of health behavior [3], as it is the most widely used psychological theory for understanding human behavior [15]. Through the lens of SCT, researchers can explore the influential and determining factors of human behavior [16]. In particular, SCT is useful in understanding how individual behavior is changed or modified [17]. SCT has been applied to predict and explain health-related behavior, in addition to human behavior in various other situations. For example, researchers have used SCT to explore determinants of individual behavior in health promotion [15], development and evaluation of physical activity interventions [18], and disease prevention [19]. More recently, studies have applied SCT to explain the determinants of health technology use behavior [20,21]. These studies using SCT were able to explain why people participate or do not participate in health-enhancing or health-damaging behavior [18]. In sum, SCT can explain the mechanisms that drive the determinants of health behavior, including health technology use, and how they connect with health practice [22]. Therefore, SCT is a useful research framework for encouraging individuals to better manage their health using health technology services.

**Figure 1:**
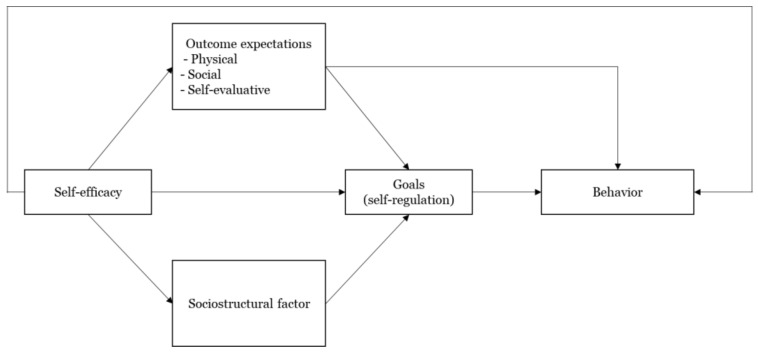
SCT of health behavior [3].

The SCT of health behavior has four constructs—self-efficacy, outcome expectations, goals (self-regulation), and socio-structural factors—which influence health behavior and promote health behavior change [3]. Figure 1 illustrates the constructs and interrelationship among them. First, self-efficacy is an individual’s belief that they can accomplish certain goals with the available resources in various situations [3,23]. A large body of research supports that self-efficacy is the primary determinant of human behavior [3,16]. In SCT, self-efficacy influences behavior directly and indirectly through other factors of SCT: outcome expectations, goals, and socio-structural factors. Second, outcome expectations are defined as an individual’s judgments about the consequences that may result from performing (or not performing) a specific behavior [17]. SCT assumes that people will act in ways that lead to more positive and valuable outcomes and avoid behaviors that lead to unfavorable outcomes [24]. Therefore, we focus on positive outcome expectations, meaning individuals’ subjective perceptions of the potential gains that will result from performing a particular behavior [25,26]. Third, goals are important because they direct changes in personal behavior. However, goals are not achieved automatically. In order to attain goals, it is necessary to have self-regulatory capability, which directs individuals’ behaviors and thoughts to accomplish a specific task despite obstacles [27]. Self-regulation differs from self-efficacy; for example, even if an individual has the self-efficacy to use health technology, they might not continuously use it in the absence of the capability to regularly perform the relevant health behaviors. Self-regulatory behavior includes three steps: first, setting individuals’ own goals; second, monitoring behaviors to achieve these goals; and third, evaluating the behaviors [1,28]. According to SCT, self-regulatory behavior is influenced by self-efficacy, outcome expectations, and socio-structural factors, and has a direct influence on behavior. Last, socio-structural factors include a variety of barriers to or facilitators of performing a certain behavior. In particular, facilitators and barriers include environmental and personal factors, which are influenced by self-efficacy and have effects on goals [18,29]. 

### 1.2. Prior Studies on SCT Regarding Health Behavior 

Due to the importance of social cognitive factors in health behaviors, SCT has been applied to the examination of health behaviors and health technology usage behavior. However, many studies have integrated SCT with other theories, and only the effects of specific determinants of SCT have been analyzed [23]. We attempted to review prior studies that examined the full model which includes all constructs of SCT. 

Firstly, we reviewed studies that applied the full SCT model to explain the health behavior of older adults. For example, in one study, the full SCT model was applied to explain the physical activity of middle-aged and older adults, and the model was tested by surveying 272 individuals aged 50 to 75 years [23]. The results demonstrate that self-efficacy, outcome expectations, and self-regulatory behavior had positive effects on physical activity, while perceived barriers had a negative effect. In addition to the four SCT constructs, the positive effects of social support were also explored. However, age, gender, and health conditions did not appear to have direct effects on physical activity. In another study, an 18-month prospective examination was conducted on whether all SCT constructs explained the physical activity behavior of older adults [30]. In total, 227 older adults with an average age of 63.8 participated in the investigation. The results show that self-efficacy influenced physical activity behavior directly and indirectly through outcome expectations, goals, and disability limitations at both baseline and 18 months. Additionally, the examination found that physical outcome expectations directly affect physical activity at both baseline and 18 months, while social outcome expectations affect physical activity only at 18 months. The study found that self-efficacy is a strong predictor of physical activity behavior in older adults, although the level of self-efficacy decreases after 18 months. However, the study also emphasized the contribution of other SCT constructs, particularly outcome expectations.

Next, we planned to review literature examining the effects of all SCT constructs on the health technology usage behavior of older adults, but there was no study examining the full SCT model. Thus, we instead reviewed a study targeting the general population. Wu et al. (2021) applied the SCT framework to understand intention to accept telemedicine in China [31]. They investigated the effects of individual factors (motivation, self-rated health, self-efficacy) and environmental factors (subjective norms, institution size, trust) on behavior intentions. However, although these researchers used SCT for their research framework, the research model did not contain the four constructs of SCT.

These studies demonstrated that some constructs of SCT have been widely applied to explain health behavior, but there are few studies which examined the full SCT model. To the best of our knowledge, no study has investigated the effect of all comprehensive components of SCT on the intentions behind continuous health app use among older users.

### 1.3. Research Model 

**Figure 2:**
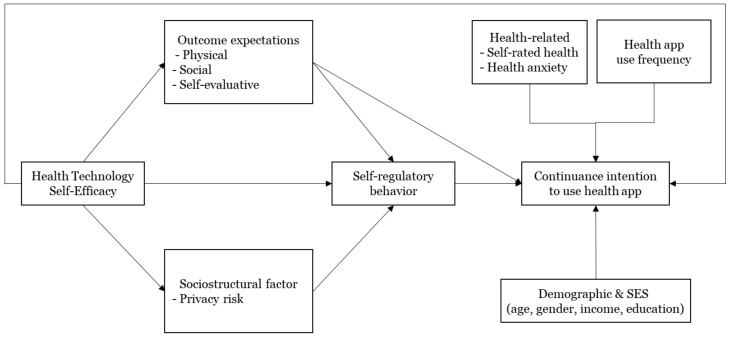
Research model.

To address the research gap established in the previous section, we applied the full SCT model as a research framework to understand older adults’ health behavior with regard to the continuous use of health apps. The research model is shown in Figure 2, and we expected that the four constructs of SCT would have direct or indirect effects on older adults’ continuous intention to use health apps. In addition to SCT constructs, health-related factors, use frequency of health apps, and general information of respondents were also expected to affect continuance intention. In this research, the term “older users” refers to users aged 60 to 79 years, as the usage of digital tools (e.g., the Internet, smartphones) is markedly different among individuals over 60 years old compared with those under the age of 60. In Korea, Internet and smartphone usage rates are close to 100% for individuals under 60 years old, while the usage rate drops to 90% for individuals in their 60s and declines steeply to 40% for those over the age of 70 [32]. Therefore, the subjects of this study are users in their 60s and 70s who are currently using health apps.

#### 1.3.1. The Effects of SCT Constructs 

Firstly, we hypothesized that self-efficacy has direct and indirect effects on the continuance intention to use health apps of older adults. We attempted to investigate the effect of self-efficacy specific to health app usage rather than self-efficacy in a general situation, because task-specific self-efficacy is more useful than general self-efficacy in maximizing the prediction of behavior in certain domains [16]. This research therefore used healthcare technology self-efficacy (HTSE), which refers to an individual’s perception of their ability to use technologies to access healthcare services [33]. The HTSE of this research is defined as users’ perceptions of their capability to use health apps without difficulties. Since HTSE is a relatively new concept compared with general self-efficacy, there are few studies on the relationship between HTSE and continuous health app use. For example, Hasa (2020) found that HTSE had a direct effect on intention to use mental health apps [34]. Apart from that study, Vinnikova et al. (2020) revealed a significant effect of technology self-efficacy on the acceptance intention and use behavior of a fitness app [35], and Gowin et al. (2019) explored the relationship between health self-efficacy and activity tracker usage in a qualitative study [36]. 

Secondly, we hypothesized that outcome expectations are influenced by self-efficacy and affect older adults’ continuance intention to use health apps either directly or indirectly through goals. The term outcome expectations in this study refers to the benefits that users can obtain from using a health app. Despite the importance of outcome expectations, they were measured in many studies using only single-dimension scales due to difficulties in measuring complicated concepts of outcome expectations [37]. Thus, we intend to examine various aspects of outcome expectations in the context of health apps. Outcome expectations are composed of various relevant outcomes related to the target behavior [3]. The subdomains of outcome expectations in the health context are physical, social, and self-evaluative, which are relevant but conceptually independent [37]. Physical outcome expectations are the judgment of physical experiences caused by health behavior, such as physical activity. In the context of health apps, physical outcome expectations refers to the expectation of health app users that the health apps will be useful in managing and enhancing their physical health status. Social outcome expectations reflect the belief of individuals that they will be more socialized and receive social approval as a result of engaging in health behavior. In this research, social outcome expectations mean that users expect to be more likely to interact with family and friends and attain stronger social approval by using a health app. Finally, the term ‘self-evaluative outcome expectations’ refers to the belief of users that they will experience satisfaction, self-worth, and healthier lives as a result of engaging in health behavior, such as health app use. Few studies have examined the influences of outcome expectations on health app use behavior. For example, Lim and Noh (2017) found that outcome expectations for exercise had a positive effect on the intention of university students to use a fitness app [38], and Park et al. (2018) also found the positive effect of outcome expectations on intention to continue using fitness apps of university students [39]. For older adults, the relationship between physical exercise and outcome expectations was clarified [40]. Gothe (2018) found that outcome expectations directly predicted the physical activity of older adults by surveying 110 African Americans with a mean age 64.77 [40]. Although these earlier studies presented valuable research results, they did not examine the effect of outcome expectations on the intention to continue using health apps of older adults and did not reflect the three dimensions of outcome expectations.

Thirdly, we hypothesized that self-regulatory behavior positively effects continuance intention to use health apps. Self-regulation is essential to achieving the goals of individuals through directing the behavior and thoughts of these individuals, leading them to participate in health behavior. However, this has not been studied as thoroughly as self-efficacy [41]. However, in some situations, the effect of self-regulation on health behavior is greater and more important than that of self-efficacy [41]. For example, engaging in moderate exercise does not require strong self-efficacy. Instead, when individuals have self-regulatory strategies to undertake regular exercise, they are more likely to engage in moderate exercise than those with strong self-efficacy [41]. In the case of health apps, such apps usually come pre-installed on smartphones, and users’ activities, such as steps, are tracked automatically without requiring any effort from the user. Thus, a high level of HTSE might not be required to use health apps. Instead, self-regulation for health behavior, such as setting one’s own exercise goals, monitoring one’s exercise activities, and evaluating one’s exercise results might have a stronger influence on continuous use of health apps. Only some studies attempted to reveal the role of self-regulation in health app usage behavior. For example, Rovniak and colleagues found that a goal setting process of self-regulation is an influential factor in fitness app use [28], and Zahrv and colleagues (2016) stated that diet apps which should embrace the three processes of self-regulation often do not incorporate all three processes [42]. 

Lastly, we attempted to clarify the effect of barriers, which are a subfactor of socio-structural factors on the self-regulatory behavior and intention to continue using health apps of older adults. SCT proposes that socio-structural factors do not directly affect behavior, but privacy risk, which is a socio-structural factor in this study, has been researched in regard to how it affects health app usage. Thus, the relationship between privacy risk and continuance intention to use health apps needs to be examined. In the field of health technology, it is critical to protect personal health information; people are anxious about exposing their personal health information. Thus, information security has been focused on as a major barrier to using health apps [43]. For example, users are concerned about unauthorized access to and unintended secondary use of their electronic health information. This could negatively influence their continuous use of health apps [43]. Therefore, we hypothesized that the information security barrier indirectly affects continuance intention through self-regulatory behavior.

#### 1.3.2. The Effects of Health-Related Factors, Health App Usage Behavior, and Demographics 

Besides the SCT constructs, the influences of other factors on continuance intention of health app use should also be considered, such as health-related factors (e.g., self-rated health and health anxiety), health app usage frequency, demographics, and socioeconomic status. It is expected that these factors are related to the continued use of health apps among older users. Previous studies have investigated the effects of these factors in the health technology context. The effect of self-rated health on health technology usage behavior has been found to be inconsistent, ranging from positive to negative to insignificant depending on the research subjects or the country in which research is conducted (see, e.g., [44,45,46]). Health anxiety refers to the fear of having a serious illness. Related research demonstrated that when individuals have stronger health anxiety, they use health technology more [47]. As demographics and socioeconomic status are predictors of the digital divide, their effects should be explored in the health app context to prevent the digital divide in the health technology field. Since these factors are relevant to health technology usage [48], their effects on continuance intention to use health apps should be explored in this research.

## 2. Materials and Methods

### 2.1. Participants and Procedures 

An anonymous online survey of 60–79-year-old respondents using health apps was conducted. The data were collected by a professional online research company who sent an email to their online research panel. The respondents were selected using quota sampling by age, gender, and place of residence to represent a general demographic composition similar to that of the broader Korean population. The response rate was 22.8% and a total of 250 respondents participated in the survey. The response rate was not high, because the inclusion criteria for recruiting respondents in this study was users of health apps, so non-users were not able to continue the survey and dropped out. The maximum margin of sampling error was ±6 percent at a 95 percent confidence level. The respondents who completed the questionnaire were remunerated with USD 10. We received consent from all respondents and clearly stated at the beginning of the online survey that the data would be used only for analysis purposes.

### 2.2. Measures 

The participants responded to 46 measures: (1) a questionnaire related to SCT, (2) health status, (3) health app use behavior and continuance intention, and (4) basic demographic information. The detailed measurement items are presented in Appendix A (Table A1).

#### 2.2.1. Measures of SCT Constructs

The measurement items related to SCT consisted of four sections: self-efficacy, outcome expectations, self-regulation, and socio-structural factor (privacy risk).

Firstly, because self-efficacy in this study was related to specific self-efficacy in the health app domain, HTSE scales were used to measure self-efficacy for using health apps. We adopted HTSE measurement items used by Rahman et al. (2016) and applied the HTSE measure to health app usage [33]. 

Secondly, the scales for three types of outcome expectations were developed based on literature about health behavior and technology acceptance behavior. The concept of physical outcome expectations is similar to usefulness in health technology acceptance research, defined as individuals’ perceptions that health technology can keep or enhance their health status [49]. Therefore, the physical outcome expectations of this study were measured based on the perceived usefulness scale items of the health app acceptance and use behavior literature [35,50]. The social outcome expectations of this study were measured using the Multidimensional Outcome Expectations for Exercise Scale, which was developed to measure outcome expectations among older adults [37]. The self-evaluative outcome expectations are in line with the perceived health outcomes, which found that technology usage in healthcare may increase user engagement and favorable responses, such as satisfaction [47]. Thus, this study measured the self-evaluative outcome expectations towards continued health app use by older users based on scales of perceived health outcomes [47]. 

The third subsection of the SCT questionnaire involves self-regulatory behavior. We used a measure of self-regulatory behavior from the literature [23,28]. They proposed the Exercise Goal-Setting Scale (EGS), which contains 10-item subscales that measure behavior related to goal development, self-monitoring, and problem-solving. Since self-regulatory behavior in this research is defined as self-regulatory ability for health behavior, not self-regulatory capability for health app usage, the original EGS measurement items were used without applying them to the health app context.

Lastly, in this study, users’ perceptions of the risks related to exposing their health-related data to others were considered a barrier to health app use. To measure the perception of such privacy risk, measurement items were developed from related earlier studies [25,43,51].

#### 2.2.2. Measures of Health-Related Factors, Health App Usage Behavior, and Demographics

In addition to the SCT-related scales, we enquired about the health status, health anxiety, health app use behavior and continuance intention, and basic demographic information of respondents. Health status was measured by asking respondents to individually assess their own health [52]. The health anxiety scale measured the degree to which respondents were concerned about their health and disease [53]. The frequency of health app usage and intention to continuously use a health app were then measured [54], and lastly, the age, gender, income, and education level of respondents were collected.

## 3. Results

SPSS Statistics version 25 (IBM Inc, Endicott, NY, USA) was used to analyze respondents’ basic information (i.e., demographics and socioeconomic status). SmartPLS (Partial least squares) version 3.3.3 (SmartPLS GmbH, Bönningstedt, Germany) was used to evaluate the measurement and structural models.

### 3.1. Demographics of Respondents 

Descriptive statistics of respondents’ demographic information are presented in Table 1. A total of 250 responses were analyzed. Respondents’ ages ranged from 60 to 79 years old, with an average of 67 years. Among all respondents, the proportion of women was higher than that of men, which is similar to the broader gender distribution of Koreans in their 60s and 70s [55]. Regarding monthly income, the most frequent responses were USD 2568~3422 (3 million~4 million Korean won). In regard to the highest level of education, the highest level of education for 42.4% of respondents was a university degree, while for 38.8% the highest level was a high school diploma. These income and education levels are slightly higher than the average for Koreans in their 60s and 70s [55], but considering the results of previous studies finding that income and educational background influence acceptance intentions of health technologies such as health apps (apart from continued use) (e.g., [46,56]), our sample can be regarded as adequately representative.

### 3.2. Evaluating the Measurement Model 

To examine the measurement model of the proposed research model, we evaluated internal consistency reliability, convergence validity, and discriminant validity.

First, to evaluate internal consistency reliability, we calculated composite reliability and Cronbach’s alpha. Variables with an outer loading less than 0.7 were excluded from the analysis [57]. Four out of 10 items measuring self-regulatory behavior, two out of four items measuring continuance intention, and one out of three items measuring health anxiety were deleted. Therefore, a total of 38 measurement items were used for analysis. The composite reliability and Cronbach’s alpha of each construct exceeded 0.7 (Table 2), indicating that the model’s internal consistency reliability was acceptable. Second, the average variance extracted (AVE) was calculated to assess convergent validity. The AVE of all constructs was greater than 0.50 (Table 2), indicating that the convergence validity is acceptable [58]. Third, to examine discriminant validity, we evaluated whether the square root of the AVE was greater than the inter-construct’s correlations [58]. As shown in Table 3, the discriminant validity of the model is also satisfactory.

### 3.3. Evaluating the Structural Model 

The structural model was examined to specify relationships among the constructs. We followed evaluation procedures for PLS-SEM (Structural Equation Modeling) reflecting the prediction-oriented nature of it. The procedures include evaluating the coefficient of determination (R^2^), cross-validated redundancy (Q^2^), and statistical significance of path coefficients [57,59].

Before evaluating structural relationships, multicollinearity between variables was evaluated using variance inflation factors (VIFs). The VIFs of the structural model were below the suggested threshold of 5, indicating no multicollinearity [57]. To assess the explanatory power of the structural model, we analyzed the adjusted R^2^ values of the six endogenous constructs (physical, social, and self-evaluative outcome expectations; self-regulatory behavior; privacy risk, and continuance intention). The adjusted R^2^ values of physical and self-evaluative outcome expectations, self-regulatory behavior, and continuance intention ranged from 0.362 to 0.476, which is moderately satisfactory. The adjusted R^2^ values of social outcome expectations and privacy risk were below 0.25, indicating weak explanatory power. Another means to examine predictive accuracy of PLS model is to calculate the Q^2^ values. As the endogenous constructs had Q^2^ values greater than zero, the model’s predictive accuracy was deemed acceptable. The R^2^ and Q^2^ values of endogenous constructs are illustrated in the Table 4. 

**Figure 3:**
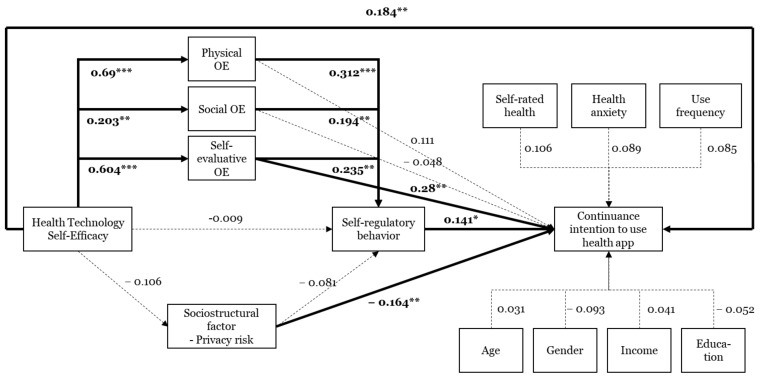
Results of structural model analysis. * *p* < 0.05. ** *p* < 0.01. *** *p* < 0.001. OE: Outcome expectations.

To examine the path coefficients, we undertook a bootstrapping procedure. Figure 3 illustrates the path coefficients, t-values, and significance levels. The factors positively influencing continuance intention to use health apps include HTSE, self-evaluative outcome expectations, and self-regulation. Privacy risk had a negative effect on continuance intention. Additionally, respondents who were male, healthier, and more frequently used health apps were more likely to continuously use health apps, although the significance level was only 0.1. We also found that physical, social, and self-evaluative outcome expectations were predictors of self-regulation. Last, we found that the three types of outcome expectations were influenced by HTSE.

## 4. Discussion

In this section, we discuss the main results reported in the preceding section. Discussion points include the role of self-regulation and outcome expectations in encouraging continued health app use among older users, antecedents of self-regulation, and the effect of respondents’ general and demographic information on their intention to continue using health apps. Based on these discussion points, we present academic and practical implications throughout this section.

### 4.1. The Important of Self-Regulation 

The results show that self-regulation—that is, the capability to direct health behavior, such as physical activity, despite obstacles—is necessary to encourage continued health app use by older users. However, it is not easy to self-regulate health behavior. People need a self-regulatory mechanism that embraces goal setting, monitoring, and the receipt of feedback. When this self-regulatory mechanism works well, self-management of the user’s health becomes possible. Before the advent of digital technologies, the self-regulatory mechanism operated on willpower alone. This meant that individuals had to record their health behavior by themselves (e.g., writing down how many steps they took and how long they slept), monitor changes in their health behaviors, and evaluate their performance. This self-regulatory mechanism was very cumbersome, and individuals usually did not engage in it continuously. Health apps can enable users to engage in this self-regulatory mechanism for health behaviors such as activity, sleep, and eating. 

Since it is important for seniors to continuously perform health behaviors, the implementation of a self-regulatory mechanism through health apps is necessary for this population. However, as the current health apps mainly target younger generations, self-regulatory strategies have been developed for them. To encourage older users’ continuous use of health apps and effective and efficient health management in the long term, self-regulatory strategies that account for physical and cognitive aging should be reflected in health apps. Additionally, considering the effects of physical, social, and self-evaluative outcome expectations on self-regulation, the self-regulatory mechanism should reflect these three benefits. For example, in the goal-setting process, goals for health behaviors should be set automatically based on the user’s age and health status. In addition, health apps should allow older adults to easily set their own goals. It is desirable that the goals should show what physical advantages and outcomes might be attained by achieving the goals. (i.e., physical outcome expectations). The second step of the self-regulatory mechanism (i.e., self-monitoring) is the most desired feature among health app users [60]. It is helpful for users to see trends in their health behavior over the course of a few days, weeks, or months so that users can monitor and control their behavior by themselves. In the case of older adults, user interfaces that show their health behavior should be developed carefully in consideration of changes in their physical and cognitive abilities. For instance, the shape, font, and color of graphs and figures should be configured to accommodate the deterioration of older adults’ visual ability, and those that are easy to understand at a glance should be provided given the decline in their cognitive resources. In addition, it is also necessary to show the health behavior of others in age groups similar to that of the older users, or to provide an opportunity to communicate with others, so that older users can feel social approval and social recognition (i.e., social outcome expectations). In the evaluation and feedback process, in order to praise and encourage health behavior, feedback notifications or messages should be provided for older adults. The feedback should emphasize older users’ achievement and self-worth maintaining or improving their health behavior (i.e., self-evaluative outcome expectations) rather than only illustrating figures, such as how many steps they walk and how many calories they consume. Sending this feedback to family members, caregivers, and doctors enables it to be used as a reference for long-term health care, and may enhance user satisfaction.

The results of this study emphasize that these outcome expectations indirectly influence the intention of older users to continue using health apps through the self-regulatory mechanism. In particular, physical and social outcome expectations do not directly affect continuance intention. This means that simply displaying physical and social outcome expectations cannot motivate older users to continue using health apps. Therefore, health apps need to be specifically designed to reflect physical and social outcome expectations in self-regulatory mechanisms. In sum, we found that the self-regulation of health behavior significantly influences continuance intentions of health app users over 60. Their capability to regulate themselves to engage in health behavior can drive continued use of health apps. At the same time, health apps have a useful function as self-regulatory mechanisms. This means that, when older users are able to set, monitor, and evaluate their health goals and behavior more easily and conveniently by using health apps, they continuously use health apps and engage in health behavior. Therefore, implementing self-regulatory mechanisms in health apps that reflect the special needs and characteristics of older adults is critical.

### 4.2. Insignificant Relationship between Self-Efficacy and Self-Regulation 

According to SCT, the antecedents of self-regulation are self-efficacy and outcome expectations. However, we found that self-efficacy (HTSE in this research) did not influence self-regulation. This is inconsistent with the core idea of SCT, which states that individuals are more likely to self-regulate health behavior when they have strong health self-efficacy. Since self-efficacy in this study referred specifically to self-efficacy in the context of health technology, it may not have influenced self-regulation. However, considering that, in Vinnikova et al.’s (2020) study [35], even self-efficacy for health apps had an effect on self-regulation, it seems that the insignificant effect of self-efficacy on self-regulation in this study was not due to the type of self-efficacy. Rather, the reason might be the respondents’ age. Young people are somewhat accustomed to managing their health through health apps, so if they are confident that they can use the health app well, this leads to the capability to regulate their health well [61]. In contrast, older users are relatively less familiar with managing their health through health apps because they lack experience in using health apps [62]. So even if they have a high level of HTSE, which is the ability to use the technology itself of the health app, they might be not aware that the health app is a useful tool for health management [63]. Instead, they may recognize the health app as only one of many apps installed on their smartphone and think of it as an app for others rather than themselves [64,65]. Thus, older users’ confidence in using health apps does not lead to their capability to regulate their health behaviors. Therefore, it is necessary to strengthen awareness among older users that the health app is a healthcare service that can help them more actively engage in healthy behavior and have better health outcomes. In addition, although HTSE did not affect self-regulation in this study, it is an important factor influencing the continued health app use among older users. Thus, it is necessary to strengthen HTSE by providing education and training programs so that older adults can use the health app without difficulty [66]. These programs should not only teach older users how to use health apps, but also teach them how to implement self-regulatory mechanisms for health behavior through health apps. 

### 4.3. Effects of Three Dimensions of Outcome Expectations 

Among the three dimensions of outcome expectations in this study, only self-evaluative outcome expectations influenced the continuance intention to use health apps. Physical and social outcome expectations did not appear to have significant effects, which is consistent with the findings in the literature that not all dimensions are significantly related to health behavior and only certain dimensions may be related, depending on the subject of the study [30]. However, we do not interpret the above finding to mean that physical and social outcome expectations are unimportant; it has been proved that these outcome expectations significantly contribute to the health behavior of older adults [30]. Rather, we suggest that as individuals age, the role of self-evaluative outcome expectations becomes more important and salient in continuance intention to use health apps. It can be seen that older users desire more emotional outcomes, such as satisfaction or self-worth, rather than realistic outcomes that physical health status improves in the functional value dimension through health app usage. This finding can be supported by research that found that when people perceive that the amount of time they can enjoy as they age is limited, they pay more attention to emotionally meaningful goals than knowledge-related goals, such as achieving new goals [67].

Despite prior studies finding significant influences of physical and social outcome expectations, the reason that physical outcome expectations were not significant in this study may be because younger adults are typically more likely to have a stronger focus on physical outcome expectations [68]. The insignificant effect of social outcome expectations is in line with results of a prior study, in which older adults did not expect social networking benefits from participation in physical activity [69]. However, studies on the health behavior of individuals with disease (e.g., breast cancer, diabetes, longstanding multiple sclerosis) found that social outcome expectations significantly influenced their health behavior [70,71,72]. This suggests that the influence of social outcome expectations may vary depending on individuals’ health status. For people with poor health, social support should be incorporated into health intervention and program design to encourage them to engage in healthy behavior [30]. The subjects of this study were in good health (80% of the participants responded with a health status which is fair, good health, and very good health), and therefore may not be interested in the benefits of social support or social approval involved in using health apps. However, we do not interpret the findings of this research to mean that social outcome expectations are not important, as the effects of social outcome expectations have been proved in various studies.

### 4.4. The Roel of Health Status, Health App Usage Frequency, and Basic Information 

In addition to factors related to SCT, the effects of health-related factors, health app usage frequency, and demographic and socioeconomic status on intention to continue using health apps were examined. As reported above, healthier respondents, more frequent users of health apps, and men showed higher intentions to continue using health apps. 

Firstly, the stronger intention to continue using health apps among older users who rated their health status as good conflicts with the research results showing that in the case of traditional medical services, the lower the subjective health status, the more health services they use [73]. However, this is consistent with the results of other studies in the fields of health technology, which show that people with good health showed more positive behavior toward health technology than those with poor health [74,75]. The association between good health status and stronger intention to continue using health apps may be explained by the mobility limitation of people with poor health. When a person’s health status is poor, it is more likely that mobility is also limited, and there is less opportunity to exercise. People being inactive are less likely to spontaneously use health apps or wearable healthcare devices [76]. Therefore, even within the same health technology, different approaches and strategies are required depending on the health status of the target.

Secondly, it has been proven that the usage frequency of health technology affects continuance use behavior [77]. For example, in the case of a support system for weight loss, willingness to continue using the system was stronger among users who engage more often with the system [77]. Therefore, in order for older users to continue using health apps, frequent access to health apps should be induced. Older users can be encouraged to frequently access health apps by stimulating a sense of achievement that they are leading a healthier lifestyle by managing their health.

Thirdly, we found that men were more likely to continue using health apps. This is different from prior research findings that women use traditional health services and health technology more [74,75]. This may vary depending on the type of health technology investigated. For example, Li et al. (2020) [74] explored factors affecting long-term use of wearable activity trackers, and Hung et al. (2020) [75] found influential factors of searching online health information and communicating with doctors online. Additionally, some studies have found that gender does not affect intention to continue using a particular service or product. Rather, it should be understood that men and women prefer or consider different characteristics of the services [78,79]. Therefore, the results should not be interpreted to mean that men are more likely to continue using health apps. We are also cautious in interpretating of this finding because the significance level is 0.1.

Finally, contrary to our expectations, health anxiety did not influence continuance intention to use health apps. Therefore, it is necessary to carefully select the target userbase for the health app. For health apps that promote healthy behaviors in daily life which are the subject of our research, users with low health anxiety and good health may be appropriate. 

However, previous study has found that user perceptions and usage behaviors may differ depending on the type of healthcare technology, such as fitness or medical [80]. Based on the different perceptions and behaviors, the target, market approaches, and policies of each type should be different as well. For example, unlike our study, in the study of Gao et al. (2015), the acceptance intention of medical health technology was influenced by threat felt about health condition [80]. Therefore, health app usage behavior in a medical context should also be investigated—specifically, how to induce continuous use of health apps for targets with strong health anxiety, such as chronic disease patients or those in need of rehabilitation after surgery.

### 4.5. Practical Implications

The results of this study provide useful practical implications which encourage the long-term health app usage of older adults. Even if there are strong expectations that health technology can play an important role in older adults’ self-management of health, in reality, older adults do not actively engage in using health technology. In order to close the gap between the ideal and reality in the health technology usage of older adults, we propose strategic approaches and directions from the cognitive, environmental, and behavioral perspectives to encourage older users to continue to use health apps. In particular, we propose practical implications related to self-regulation and outcome expectations, which have received relatively little attention when compared to self-efficacy, which has been researched as the main factor of SCT.

Firstly, this study emphasizes the importance of self-regulation in the long-term use of health apps. Based on the results regarding self-regulation, we propose practical implications on how the self-regulatory mechanism should be designed to better reflect the physical, cognitive, and emotional changes of older adults due to aging. Secondly, we presented the roles that outcome expectations should perform to motivate older adults to use health apps continuously. For example, we suggested how physical, social, and self-evaluative outcome expectations can be implemented in health apps. The health apps implementing these three types of outcome expectations can provide the benefits desired by older users. Lastly, we proposed strategic approaches of health apps and stated these strategies should be different depending on the type of health app. This demonstrates the need to segment markets and strategies according to the type of health technology (e.g., medical or healthcare).

### 4.6. Limitations 

Despite the implications and contributions of this study, this study has theoretical and practical limitations.

Firstly, we did not analyze the effect of each of the three processes of self-regulatory mechanism on the intention of older adult to use health apps continuously. For example, among self-regulatory mechanisms, including goal setting, monitoring, and feedback, there may be a specific stage that has a greater impact on how older adults use health apps usage compared to that of other stages. Thus, considering the importance of self-regulation found in this study, if the relative influence of the three stages is further analyzed, a more effective self-regulatory strategy can be developed.

Secondly, the relationship between the age, education level, income level of older users and the intention to continue using the health app should be studied in more detail. For example, by conducting multigroup analysis, the effects of each variable on the intention to continue using health apps of older users could be compared among groups. These results would be useful in suggesting practical implications such as more detailed target groups and market approaches. 

Thirdly, the effects of health status, health app use frequency, and gender were discussed at the significance level of 0.1. Consequently, we were cautious in how we interpreted these effects. Once the research model of this study has been repeatedly verified, tested with different samples, or when behaviors for different health services have been studied, the effects of these factors may appear in different directions or appear insignificant.

Finally, the effects of uncovered sub-factors of socio-structural factors should be investigated. Various sub-factors from personal and environmental perspectives might influence the continuance intention of older adults to use health apps, such as the perceived trustworthiness of health app providers, health app contents [31], and how the user is affected by the judgement of others around them.

## 5. Conclusions

The objective of this study was to examine the factors that influence intention to continue to use health apps among users aged 60 to 79 years. This research developed a conceptual model based on SCT, which integrates cognitive, environmental, and behavioral perspectives. This integrated approach is essential because health technology use among older adults is a complex process involving personal, environmental, and technological factors [81]. Based on this theoretical background, we proposed the research model. Specifically, we evaluated how the constructs of SCT (i.e., self-efficacy, outcome expectations, self-regulatory behaviors, privacy risk) were related to the continuance intention of older adults to use health apps, while also considering the effects of health status, health anxiety, frequency usage, and demographics of respondents. The proposed research model was tested through an online survey. To the best of our knowledge, this research is among the first to investigate continuous health app use behavior among older adults from an SCT perspective.

There are three main contributions of this study. First, based on SCT, we were able to explore previously undiscovered determinants of continuance intention to use health apps among users aged 60 years and older. The determinants we found are differentiated from the influencing factors previously found in the literature based on technology acceptance theories. Second, the effects of self-regulation of health behaviors and the three types of outcome expectations on older adults’ continuance intention for health app use were examined in detail. Since these factors have not been studied much in the literature in relation to older people’s use of health technology, the results of this study, which examined the effects of unrevealed factors in detail, are academically significant. The first and second contributions provide practical implications, as well. For example, they can guide which functions health apps for older adults should provide and how health apps should be designed for such users. These implications can motivate older adults’ continued use of health apps, which leads to effective and efficient healthcare for older users, and ultimately demonstrate positive effects in terms of promoting health among older people over the long term. Third, in addition to the SCT constructs, we examined the effects of factors related to health, health app usage frequency, and demographics on continuance intention to use health apps, which can be helpful in defining the targets of health apps.

## Figures and Tables

**Table 1:** Demographic characteristics of respondents.

Characteristics		*N*	%
Gender	Male	119	47.6
	Female	131	52.4
Age	60~64	108	43.2
	65~69	55	22.0
	70~74	76	30.4
	75~79	11	4.4
Income (monthly)	<USD 855	23	9.2
	USD 855–1711	34	13.6
	USD 1712–2567	36	14.4
	USD 2568–3422	54	21.6
	USD 3423~4278	46	18.4
	USD 4279~5133	15	6.0
	≥USD 5144	42	16.8
Highest level of education	Middle school	11	4.8
	High school	97	38.8
	College/university	106	41.4
	Graduate school	35	14.0

**Table 2:** Reliability and convergent validity test results.

Latent Variable	Composite Reliability	Cronbach’s Alpha	AVE
HTSE ^1^	0.925	0.892	0.757
Physical OE ^2^	0.923	0.888	0.749
Social OE	0.929	0.897	0.766
Self-evaluative OE	0.895	0.820	0.742
Self-regulation	0.901	0.873	0.565
Privacy risk	0.909	0.866	0.716
Health anxiety	0.908	0.860	0.768
Continuance intention	0.912	0.809	0.839

^1^ Health technology self-efficacy. ^2^ Outcome expectations.

**Table 3:** Results of discriminant validity analysis.

	1	2	3	4	5	6	7	8
1	0.87 *							
2	0.69	0.865						
3	0.203	0.406	0.875					
4	0.604	0.75	0.403	0.861				
5	0.397	0.564	0.409	0.544	0.752			
6	−0.106	−0.041	0.052	−0.027	−0.089	0.846		
7	0.073	0.025	0.087	0.046	−0.05	0.173	0.876	
8	0.563	0.563	0.227	0.582	0.483	−0.251	0.026	0.916

* Diagonal values represent square root of average variance extracted. 1: Health technology self-efficacy, 2: physical outcome expectations (OE), 3: social OE, 4: self-evaluative OE, 5: self-regulation, 6: privacy risk, 7: health anxiety, 8: continuance intention.

**Table 4:** The R^2^ and Q^2^ values of endogenous constructs.

Latent Variable	R^2^	Adjusted R^2^	Q^2^
Physical OE ^1^	0.477	0.475	0.349
Social OE	0.041	0.037	0.029
Self-evaluative OE	0.365	0.362	0.265
Self-regulation	0.387	0.374	0.206
Privacy risk	0.011	0.007	0.006
Continuance intention	0.503	0.476	0.382

^1^ Outcome Expectations.

## Data Availability

Data available on request due to ethical restrictions.

## Appendix A. Measurement Items

**Table A1:** Measurement items of the constructs.

Construct	Measurement Items	References
Health technology self-efficacy	It is easy for me to use health apps.	[33]
	I do not feel comfortable using health apps (reverse coded).	
	I have capability to use health apps.	
	I would be able to use health apps without much effort.	
Physical outcome expectations	Health apps improve my performance in managing my physical activity.	[35,50]
	Health apps increase my productivity such as more physical activity.	
	Health apps enhance my effectiveness in physical activity.	
	Overall, health apps are useful in managing goals related to physical activity.	
Social outcome expectations	Using health apps provides companionship.	[37]
	Using health apps makes me more at ease with people.	
	Using health apps increases my acceptance by others.	
	Using health apps improves my social status.	
Self-evaluative outcome expectations	Using health apps has helped me to lead a healthy satisfied life	[37,50]
	Using health apps has helped me to maintain a good healthy satisfied life.	
	After using health apps, I feel a sense of achievement regarding having a healthy life.	
Self-regulatory behavior	I often set exercise goals (deleted).	[23,28]
	I usually have more than one major exercise goal (deleted).	
	I usually set dates for achieving my exercise goals.	
	My exercise goals help to increase my motivation for doing exercise.	
	I tend to break more difficult exercise goals down into a series of smaller goals (deleted).	
	I usually keep track of my progress in meeting my goals.	
	I have developed a series of steps for reaching my exercise goals.	
	I usually achieve the exercise goals I set for myself.	
	If I do not reach an exercise goal, I analyze what went wrong.	
	I make my exercise goals public by telling other people about them (deleted).	
Privacy risk	I am worried when I think that by using health apps my personal information might be exposed to.	[25,43,51]
	Using health apps interferes with my life by providing unnecessary advertisements or information.	
	Using health app would enable third-party to misuse my personal data.	
	Overall, I see a privacy threat linked to health app usage.	
Health anxiety	I am afraid that I have a serious illness.	[53]
	I worry about my health (deleted).	
	If I hear about an illness, I think I have it myself.	
Continuance intention to use health app	I intend to continue using health apps rather than discontinue their use.	[7,54]
	My intentions are to continue using this health app rather than to use any alternate means, such as pedometer (deleted).	
	I prefer to use health apps again	
	If I could, I would like to discontinue my use of health app (reverse coded), (deleted).	
